# iRODS metadata management for a cancer genome analysis workflow

**DOI:** 10.1186/s12859-018-2576-5

**Published:** 2019-01-15

**Authors:** Lech Nieroda, Lukas Maas, Scott Thiebes, Ulrich Lang, Ali Sunyaev, Viktor Achter, Martin Peifer

**Affiliations:** 1 Regional Computing Center (RRZK), University of Cologne, Cologne, 50931 Germany; 2 Department of Translational Genomics, Center of Integrated Oncology Cologne-Bonn, Medical Faculty, University of Cologne, Cologne, 50931 Germany; 3 Department of Economics and Management, Karlsruhe Institute of Technology, Karlsruhe, 76133 Germany; 4 Center for Molecular Medicine Cologne (CMMC), University of Cologne, Cologne, 50931 Germany

**Keywords:** Next generation sequencing (NGS), Genome analysis, iRODS, Workflow integration, High performance computing (HPC), Data security, Data consistency, Metadata management

## Abstract

**Background:**

The massive amounts of data from next generation sequencing (NGS) methods pose various challenges with respect to data security, storage and metadata management. While there is a broad range of data analysis pipelines, these challenges remain largely unaddressed to date.

**Results:**

We describe the integration of the open-source metadata management system iRODS (Integrated Rule-Oriented Data System) with a cancer genome analysis pipeline in a high performance computing environment. The system allows for customized metadata attributes as well as fine-grained protection rules and is augmented by a user-friendly front-end for metadata input. This results in a robust, efficient end-to-end workflow under consideration of data security, central storage and unified metadata information.

**Conclusions:**

Integrating iRODS with an NGS data analysis pipeline is a suitable method for addressing the challenges of data security, storage and metadata management in NGS environments.

## Background

**Fig. 1:**
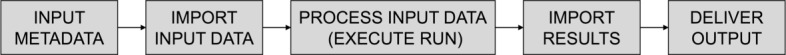
High level end-to-end workflow Values for predefined ‘input’ metadata attributes are prepared per NGS data sample. The NGS data is moved to central and secure storage (vault) and tagged with the metadata values. To process the NGS data a ‘run’ of the HPC analysis pipeline is executed, whose results are again moved into the vault and tagged with additional metadata values of the run. Finally, the results of the run are delivered to the data owner

Next generation sequencing (NGS) is an increasingly cost efficient and reliable method to provide whole genomes or exomes (i.e., the protein coding part of the genome) in a relatively short time. Due to falling costs it became feasible to widen the scope of sequencing research and applications, for example from assembling a single human genome in the nineties, through analysis and comparison of thousands of genomes in the last decade up to the point where personalized medicine has been partially realized. The massive amounts of resulting data pose various challenges that need to be addressed in order to enable their exploration, analysis and effective dissemination. In particular, genetic data runs through a lifecycle: the raw data, generated by high throughput sequencing machines, is organized and stored depending on its type (e.g., genome/exome) and origin, later on it is retrieved, processed and analyzed on high performance computing (HPC) infrastructures. Once the final results have been computed, these are made available for reviewing, additional processing and further comparison. This lifecycle is accompanied by at least two roles: a data owner and a data analyst. At each step of the lifecycle the correct data needs to be identified, located, and processed in a secure way. Each user access needs to be evaluated whether it is authorized and whether the proposed additions conform to the existing data schema or format. Those additions (e.g., quality control values or statistics regarding genome alignment) are usually too extensive to be added to the file name and too valuable to be dumped into a text file without adequate means of search or aggregation. Traditional file systems quickly meet their limits both in terms of fine-grained authorization as well as in terms of metadata, where content based information such as the project, sample ID, performed analysis, and results are required, as they operate with a simple username and -group principle, and possibly with more advanced access control lists to manage access. The metadata is limited to system properties like ownership, size, date of creation or modification of files and directories. Thus it becomes apparent that a more sophisticated system would be required. The present paper describes the implementation of such a system which was designed to embed the in-house NGS analysis pipeline (see “NGS data analysis pipeline” section) into an end-to-end workflow utilizing the comprehensive data management system iRODS (Integrated Rule-Oriented Data System) [1] and a web-based in-house developed front-end for metadata input and workflow management. Based on our experiences with NGS workflows in HPC environments [2, 3], we have decided to use iRODS since it allows for customized metadata attributes, fine-grained protection rules as well as a query system to quickly organize and review the results of a cancer genome analysis workflow. The high level workflow is depicted in Fig. 1.

### iRODS (Integrated rule-oriented data system)

The most prominent feature of the data management system iRODS is the possibility to add customized metadata to all stored files, thus allowing researchers to track and manage their original input and results. In our implementation this includes descriptions of sample origins (e.g., project, patient and specimen identifier, cancer type, and tissue information), analysis pipeline processing options (e.g., an application use case described in “Efficient use of storage and computing resources” section) as well as the actual processing results. All of these metadata can be subsequently searched via SQL-like queries, either by using the iRODS command line tools (iCommands) or one of the add-on graphical user-interfaces such as the iRODS Cloud Browser [4]. In addition, the Cloud Browser offers a convenient interface to browse through directory structures, to view and to edit metadata, and to download files, which may be even simpler for less experienced users. Overall, the system offers high flexibility through a number of different APIs and a user-friendly graphical interface as well. The system also provides a rule-engine that enables the execution of predefined actions in regular intervals, triggered by certain events or manual control. Any data operations can thus be augmented with matching actions (e.g., leaving an audit trail, submitting HPC jobs once certain data files arrive, or sending a message after particular events). iRODS uses a virtual file hierarchy that can be adapted to various organizational structures and is independent from the actual physical storage. The access control can be easily fine-tuned from encompassing groups down to single users, giving a flexibility similar to POSIX Access Control Lists, regardless of whether the underlying file system supports it.

### NGS data analysis pipeline

The NGS analysis pipeline is an in-house development that has been successfully applied to a variety of large-scale cancer genome sequencing projects [5–11]. In the first step, the pipeline aligns raw whole genome or exome sequencing data to a reference genome using bwa-mem [12]. After alignment, the data is preprocessed to allow mutation detection. To this end, alignments are sorted, indexed, and potential PCR-duplications are masked. Quality control statistics of the sequencing run (mean coverage, insert size, etc.) are computed along with other required pre-processing data for mutation calling. Subsequently from these data single nucleotide substitutions, small insertions and deletions, copy number changes, and genomic rearrangements (the latter only in case of whole genomes) are determined. Except for the alignment method, the entire set of computational methods are own developments that are uniformly implemented in C++ to allow for an efficient processing of large data sets. Many of these methods are freely available in the Sclust package [13]. To facilitate a streamlined and robust workflow, we have automated the pipeline using shell scripts and introduced error handling procedures to react to both internal content based as well as external system based errors.

It is important to note that the NGS analysis pipeline is in principal independent of the system described in the present text: here we describe the augmentation of a given analysis pipeline with metadata management using the iRODS system. However, to give the reader a better understanding of the original setting, we have included the above description of our own pipeline.

## Results

The following sections present the main ideas underlying our implementation. Our system is in production at the University of Cologne and is currently used by our department.

### Processing methods according to the data’s lifecycle

In correspondence with the observed lifecycle of genetic data (see “Background” section), we separate the *input* from *run* related methods and data. While the term *input* comprises initial import and storage of raw data, *run* means retrieval and processing of the input as well as the quality control of the results. This separation is natural with respect to multiple processing runs of the same input data. Such multiple runs occur, for example, because of multiple tumor samples of the same donor that are subsequently compared to previously processed (normal or prior tumor) samples in tumor evolution studies such as [14, 15], or because the effect of different program parameters is under analysis. Furthermore, original raw *input* data is replaced with its annotated version once a *run* is completed successfully. In any case, redundant storage or processing is avoided. Multiple, related samples are handled via simple index structures, see “Data consistency within the vault is enforced by predefined value domains” section.

### A central vault and a single user for enhanced security

Since genome sequencing provides the complete genomic fingerprint of its specimen donor, high security standards for handling such data are indispensable. To protect data transmissions en route as well as to restrict access to predefined hosts, all iRODS iCommand clients have been set up to communicate through host-certificate based SSL encryption. The iRODS server has a main vault directory, which holds the data archive and is owned exclusively by a specific irods user. All other machines have to use iCommand Clients or APIs to download, upload, and query the data. With such a setup any data located in the vault is shielded from all HPC users through locally managed file ownership and permissions. Although the security within the vault depends on the iRODS authorization mechanisms, these mechanisms add an additional layer of security and data protection compared to standard file access control mechanisms based on file permissions or access control lists. Additionally, in order to document data access operations for sensitive data collections, the iRODS audit plugin [16] has been installed and pipelined with tools of the ElasticSearch Suite [17].

### The workflow is role-based

Besides the virtual irods user, we distinguish between two further roles in the implementation of our workflow: the *data owner* who drives the collection and the sequencing of a certain cancer specimen, and the *data analyst* who is responsible for processing the workflow from the import of input data to the output of results. More precisely, as soon as the input data has arrived from the sequencing facility and the data owner has provided the input metadata, the data analyst takes over to operate *input* and *run* methods before the results are delivered to the data owner. In particular, the separation of roles allows for role-specific data access rights such as read-only access for the data owner to the processed data securely stored in the vault. While read-write access for data analysts is mandatory, in our experience the data owner is well served by accessing, for example, large (bam) files containing sequencing reads with alignment information read-only via a local mount of the vault, while smaller output (txt or pdf) files such as the analysis results of the genomic mutation calls or copy number alterations can be delivered on demand for subsequent read-write access under local use to the data owner. Thus, original analysis results remain unaltered inside the vault while the data owner is not restricted in manipulating his own copy of the data. If the data owner wants to preserve a certain state of his edited data, then it can be imported into the system and optionally tagged with additional user-defined metadata.

### Data consistency within the vault is enforced by predefined value domains

To ensure metadata consistency, both content- as well as format-wise, we have defined simple schemata for the input (data import) and the run (analysis pipeline) execution, respectively. Metadata information is provided in comma-separated value (csv) files describing the workflow step (input or run), predefined attributes and their corresponding values that must adhere to predefined value domains. These csv files are called input sheets and run sheets, respectively. In our implementation, we have designed a web-based front-end to ease input and successive generation of (batches of) such sheets which is described in “A front-end for metadata input and workflow monitoring” section. The metadata information sheets are parsed by perl scripts, which validate them and perform additional tests, if required by the schema (see an excerpt in Table 1). The attributes depend on the step of the workflow: the input sheets for importing the input data into the vault are designed to describe the data origin as thoroughly as possible while the run sheets focus on their application (e.g., the genomic reference sequence to align the data against). Some of the attributes are generated by the scripts, depending on encountered input file format, creation date and other factors.

**Table 1:** Excerpt from input and run schemata

Type	Attribute	Example value	Value domain	Further tests
Input	Local_path	/projects/user/samples	[a-zA-Z0-9_-+/.]	Path readability
Input	Filename	sample1_T.bam	[a-zA-Z0-9_-+/.].{bam}	File readability
Input, run	Sample_ID	P1234-PB03	[a-zA-Z0-9_-+.]	None
Input	Data_owner	Alice	[a-zA-Z- ]	None
Run	Use_case	2	[1-4]	None

The perl scripts create *virtual paths* in iRODS adhering to a fixed structure of certain attribute values. Thus the data is placed, and subsequently can be located, in unique locations such as:


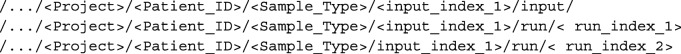


The import does only succeed if the user has sufficient rights for all components of the generated virtual path. Once the metadata has been validated, tested and extended with dynamically generated content (e.g., time stamps, checksums), it is packed into attribute-value-unit triplets and uploaded together with the input files via the iput command from iRODS’ iCommands.

### Scripts integrate iRODS with the analysis pipeline

A short overview of the NGS data analysis pipeline is given in “NGS data analysis pipeline” section. However, for the present paper it is sufficient to know that the pipeline is controlled by a shell script which processes input data subject to certain options until it exits with output data and a status (success or failure of execution). To integrate iRODS with the pipeline processing, a perl *run script* parses a run sheet and calls the pipeline control script with input data and options automatically deduced from metadata information and the vault directory structure (e.g., project, patient identifier and sample type parsed from the run sheet, and the largest corresponding input index present in the vault). Retrieval of the appropriate files is implemented with simple SQL-like queries which are executed using the iquest and iget commands. Once the pipeline control script exits successfully, the run script proceeds with uploading the results from predefined output directories into the vault; otherwise, the run script aborts and may be called again for an upload of results after the data analyst has ensured a successful pipeline execution. While uploading results into the vault, all files are tagged with run metadata as provided from the run sheet as well as additional metadata generated during pipeline processing. Moreover, ownership of the files inside the vault is changed to the irods user.

**Fig. 2:**
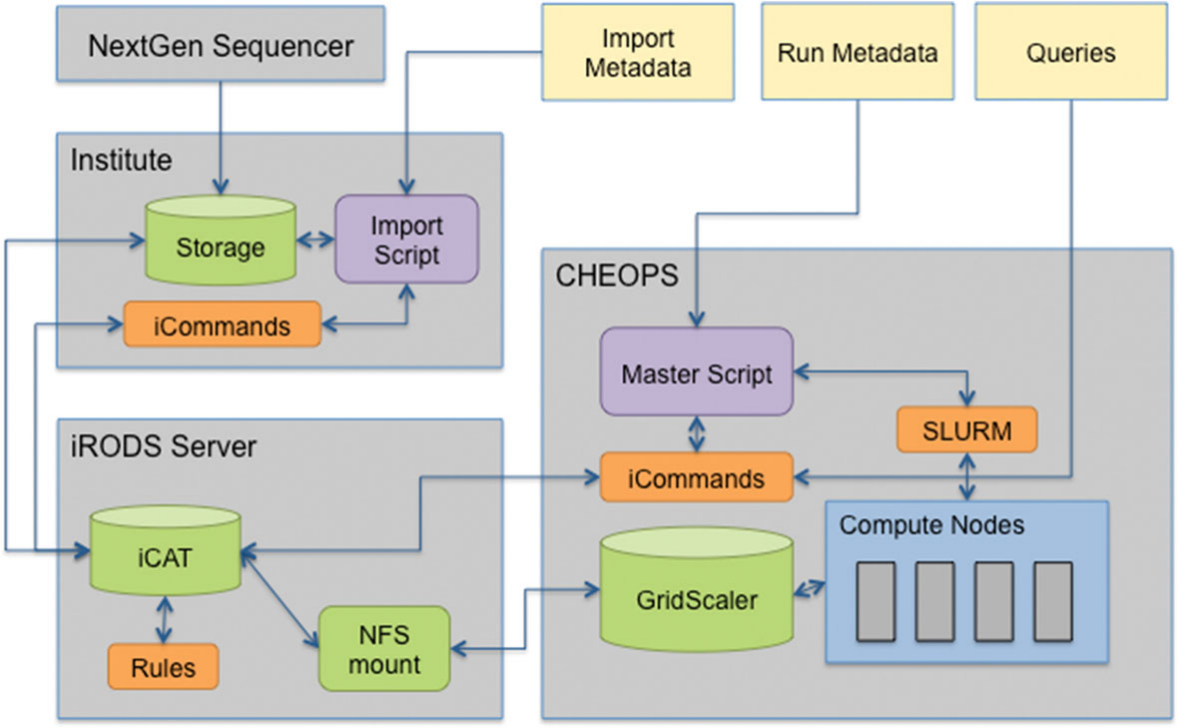
Solution architecture overview NGS data is stored on the institute’s physical storage and the input metadata is prepared. An import script validates the metadata and passes it to a virtual machine providing iRODS server resources. Up to this VM, all other machines have to use iCommand Clients or APIs to download, upload or query the data. All iRODS iCommand clients have been set up to communicate through host-certificate based SSL encryption. The iRODS Server has a main vault directory, which holds the data archive and is owned exclusively by the iRODS user. It is physically located on the GridScaler Storage System of the HPC (CHEOPS) and made available to iRODS via an NFS mount, which can also be accessed only by the irods user. In particular, we have decided to let iRODS maintain its data within the vault but restrict it to a dedicated server with stringent access policies. With such a setup any data located in the vault is shielded from all CHEOPS users through locally managed file ownership and permissions but the security within the vault depends entirely on the strength and infallibility of iRODS authorization mechanisms. To run the NGS pipeline on the HPC (CHEOPS), run metadata is prepared and a master script orchestrates the communication with iRODS to query and retrieve the input data and to import the results after successful processing

The high level solution architecture is depicted in Fig. 2.

### A front-end for metadata input and workflow monitoring

To simplify metadata input and workflow monitoring for the data owners (some of which may have limited computer literacy) and to establish a common workflow entry, independent of the data analyst, we have implemented a lightweight web-based front-end. Similar to the backend, users of the front-end are assigned the role of a data owner or data analyst. Upon logging in to the front-end, data owners can create new jobs and send them to a data analyst. Data analysts are automatically notified about the availability of new jobs within the front-end and via email. They can then process each job individually and add further required information (e.g., filenames and the virtual path to the respective genome data). After all information has been entered, data analysts can download input and run csv files to start the next phase of the workflow for each job. During the process, data owners have access to their jobs and may monitor their current states. Finally, data analysts can notify data owners about the status and, in particular, the successful completion of their jobs.

**Fig. 3:**
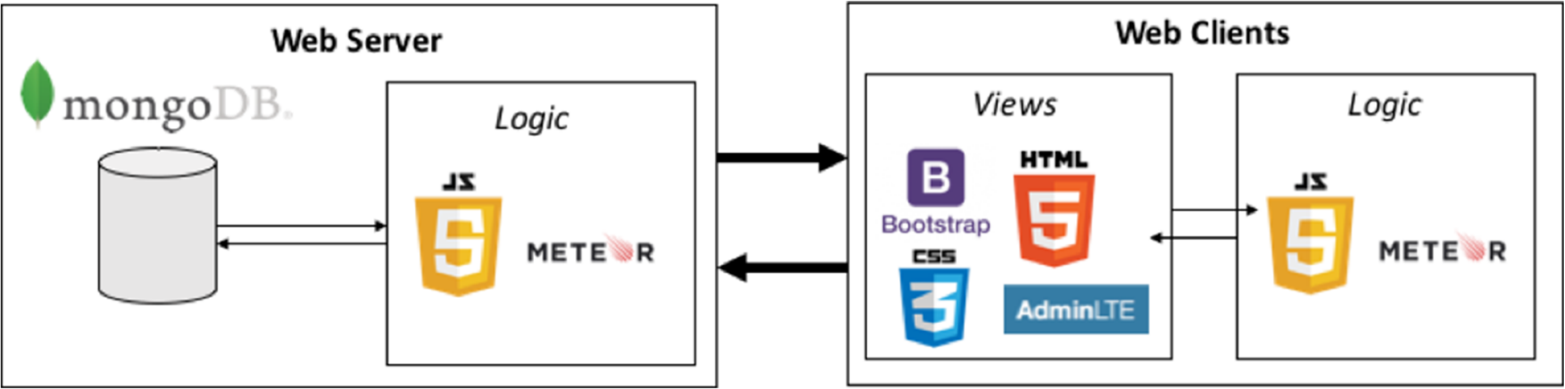
Overview of front-end architecture with frameworks/tools The web-based front-end is built on the basis of the MeteorJS framework. On the server side, MeteorJS connects to a document-based NoSQL MongoDB; on the client side, the CSS framework AdminLTE 2.0 based on Bootstrap is used

From a technical perspective, the front-end is built with MeteorJS, an open-source web framework that provides us with many ready-to-use functionalities as well as real-time communication and updates between each connected client. On the server side, MeteorJS connects to a document-based NoSQL MongoDB, which allows for easy and fast storage of large amounts of metadata. On the web-client side we use Bootstrap and the AdminLTE 2.0 framework to build an intuitive and easy-to-use user interface. Overall, this architecture allows for developing a secure, fast, and modern web front-end within a short timeframe. Furthermore, the front-end is fully customizable, meaning that metadata fields can be added or removed at runtime without adding any code and without corrupting extant jobs. Figure 3 gives an overview of the front-end’s architecture.

## Discussion

Numerous NGS workflows have been adapted to HPC systems with various methods. For example, HugeSeq [18] detects and annotates genetic variations by applying a MapReduce [19] approach, NGSANE [20] uses bash scripting with extensible logging and checkpointing measures, SIMPLEX [21] offers a fully functional VirtualBox Image to reduce installation issues. While they describe the analysis process in detail, few of them consider the requirements of data security and the necessary framework to make the results as well the corresponding metadata available for further dissemination. The WEP [22] pipeline for whole exome processing addresses the latter shortcoming by storing result metadata in a self developed MySQL database with a PHP-based web interface. What is missing is a comprehensive data management system that would encompass the employed input data, the results and the metadata within a secure and reliable framework. Even though the metadata delivers necessary information, the underlying files should also be stored in a controlled environment, so that they can be both retrieved at a moments notice.

There are organizations that have employed iRODS for their NGS workflows, namely the Wellcome Trust Sanger Institute [23], Broad Institute, Genome Center at Washington University, Bayer HealthCare, and University of Uppsala (private communication); most recently, the University of Arizona has developed a widely integrated cloud solution for NGS data processing and analysis [24] which is partly based on iRODS. However, they mainly use iRODS to manage, store and retrieve data (e.g., alignment files). In contrast, by encompassing our workflow with iRODS, we can not only store and annotate the input data with relevant information but also parse the results and make them available for queries through self-defined metadata within a single system.

The described pipeline automation and integration with iRODS empowers (organizations of many) scientists to keep track of their data in an efficient and secure manner. By employing verifiable data schemata, we can enforce metadata consistency and build a hierarchical structure within iRODS’ virtual file space that places files in predefined locations. While it provides a straightforward means to narrow data searches down, it also makes the mapping of user permissions easier to manage. The possibility to restrict access to certain projects or file groups is especially relevant in the clinical context where patient data is involved. We have decided to rely on iRODS’ authentication in order to let it manage contents in their entirety, rather than using it as a sole metadata provider. For this means we have also tightened security and restricted its services to a virtual machine as well as a resource server within the cluster. The latter leverages low latency and high bandwidth network capabilities.

The inclusion of both the input as well as output data with matching descriptions has resulted in a comprehensive system that allows to retrieve and compare analysis results with their underlying sources.

### Efficient use of storage and computing resources

A common use case in cancer genomics is to compare NGS data of tumor specimen against data of a normal tissue specimen, both collected from the same patient. Thus we often process pairs of tumor and normal data. During course of the patient’s treatment sometimes additional tumor specimen are collected, sequenced, and subsequently compared against the previously collected normal data (e.g., in order to understand the tumor evolution). As a common problem in practice, redundant copies of normal data files are made for each tumor data analysis; likewise, often multiple redundant data copies arise when different projects work on the same input data. In practice, a proper clean-up of such redundant data is time-consuming and often missing. While this can be addressed in principle by establishing organizational data storage and processing policies alone, the proper execution of such a policy is a clear benefit of our implementation of the complete workflow.

Additionally, in order to minimize the use of computing resources we distinguish between different use cases for running the cancer genome analysis pipeline: either, a pair of tumor and normal data, or new tumor data with respect to previously analyzed normal data is processed. In the latter case, processing results of the normal data are retrieved from the vault and used for the current analysis.

## Conclusions

Integrating iRODS with an NGS data analysis pipeline is a suitable method for addressing the challenges of data security, storage and metadata management in NGS environments. Collected metadata, either by input or automatic generation, can be harvested for characterizing the data repository and for optimizing processing protocols and options. Selection of the relevant metadata for such analyses is straightforward and avoids data gathering from multiple sources. Moreover, the common data structure provides the data as input for subsequent use in a quick and neat fashion. Proper archiving of the huge amounts of project data is another natural extension of our workflow. Besides, integration of other databases that collect some metadata related to sequencing data (e.g., for administrating the workflow from specimen collection until delivery of the sequencing data) may eventually lead to a single source of truth-like data store. However, due to the usual trade-off between integration efforts and the solutions usability and complexity, this may not always be beneficial.

## Methods

General system descriptions of iRODS and the NGS data analysis pipelines are given in “iRODS (Integrated rule-oriented data system)” and “NGS data analysis pipeline” sections, respectively. “Results” section describes the integration of iRODS with our NGS data analysis pipeline in detail.

## Abbreviations

API: Application programming interface
csv: Comma-separated values
HPC: High performance computing
iRODS: Integrated rule-oriented data system
NGS: Next generation sequencing
PCR: Polymerase chain reaction
POSIX: Portable operating system interface
SQL: Structured query language
